# Mortality and socio-economic outcomes among patients hospitalized for stroke and diabetes in the US: a recent analysis from the National Inpatient Sample

**DOI:** 10.1038/s41598-021-87320-w

**Published:** 2021-04-15

**Authors:** Aya Tabbalat, Soha Dargham, Jassim Al Suwaidi, Samar Aboulsoud, Salman Al Jerdi, Charbel Abi Khalil

**Affiliations:** 1 Research Department, Weill Cornell Medicine-Qatar, PO box 24144, Doha, Qatar; 2 Heart Hospital, Hamad Medical Corporation, Doha, Qatar; 3 Department of Medicine, Kasr Alainy, Cairo University, Cairo, Egypt; 4 Joan and Sanford I, Weill Department of Medicine, Weill Cornell Medicine, New York, USA

**Keywords:** Endocrinology, Health care, Neurology

## Abstract

The prevalence and incidence of diabetes mellitus (DM) are increasing worldwide. We aim to assess mortality and socio-economic outcomes among patients hospitalized for stroke and diabetes in the US and evaluate their recent trends. We examined: in-hospital mortality, length of stay (LoS), and overall hospital charges in diabetic patients over 18 years old who were hospitalized with a stroke from 2005 to 2014, included in the National Inpatient Sample. In those patients, the mean (SD) age slightly decreased from 70 (13) years to 69 (13) years (p-trend < 0.001). Interestingly, although incident cases of stroke amongst DM patients increased from 17.4 to 20.0 /100,000 US adults (p-trend < 0.001), age-adjusted mortality for those with hemorrhagic strokes decreased from 24.3% to 19.6%, and also decreased from 3.23% to 2.48% for those with ischemic strokes (p-trend < 0.01 for both), but remained unchanged in TIAs patients. As expected, the average total charges per hospital stay almost doubled over the ten-year period, increasing from 15 970 to 31 018 USD/stay (adjusted for inflation). Nonetheless, median (IQR) LoS slightly decreased from 4 (2–6) to 3 (2–6) days (p-trend < 0.001). In total, our data show that, from 2005 to 2014, the incidence of stroke among the diabetes patient population are gradually increasing, in-hospital mortality is steadily decreasing, along with average LoS. Admission costs were up almost twofold during the same period.

## Introduction

Stroke is the leading cause of long term disability^1^, and the fifth most common cause of mortality in the United States^2^. In 2016, the global lifetime risk of stroke for adults >  = 25 years was 24.9%; a relative increase of almost 9% since 1990^3^.

Over the last decade, the prevalence of diabetes mellitus (DM) has continued to rise worldwide due to the aging of the population and a pandemic surge in obesity and sedentary lifestyles^4^. Patients with DM have an increased risk of developing large- and medium vessel acute atherosclerotic disease, in addition to microangiopathy^5^. This accelerated atherosclerosis makes hyperglycemia an important risk factor for ischemic stroke, resulting in an almost twofold increased incidence in DM patients^6^. This risk is also notably higher in women^7–9^. Further, diabetes also increases the risk of hemorrhagic stroke^10^, which is majored by the concomitant presence of hypertension and uncontrolled hyperglycemia^11^.

Cardiovascular disease remains the leading cause of death worldwide despite the implementation of more efficacious primary and secondary prevention measures^12^. Nevertheless, recent temporal trend analysis suggest that CVD-related mortality is decreasing gradually^13,14^, in particular from stroke and coronary artery disease^14^. In stroke patients, it is estimated that mortality decreased by over 55% in a decade^15^.

Despite the steady increase in diabetes’ incidence and prevalence, age-adjusted and cause-specific mortality and hospitalization for some cardiovascular complications in DM patients has also been on the decline^16^. For instance, in-hospital mortality in diabetic patients hospitalized for myocardial infarction was reduced by almost 4%^17^.However, it is not known whether the presence of diabetes in stroke patients follows the same trend. We therefore assessed mortality and socio-economic outcomes among patients hospitalized for stroke and diabetes in the US and examined their national trend.

## Methods

### Database

Data were extracted from the National Inpatient Sample (NIS) database between 2005 and 2014. NIS is the largest publicly available hospital discharge database in the United States, initiated in 1998 as part of the Healthcare Cost and Utilization Project (HCUP), and funded by the Agency of Healthcare Research and Quality (AHRQ)^18^. Approximately 7 million hospitalizations are recorded every year from over 1000 hospitals, which represent 20% of all inpatient admissions to non-federal hospitals in the US. The database contains de-identified information related to diagnosis, procedures, in-patient outcomes, patient demographics, cost and disposition status^19^. The study was waived from institutional review board because participants were de-identified in the NIS.

### Diagnosis and outcomes

All diagnoses and outcomes were identified using the International Classification of Disease, Ninth Revision, Clinical Modification (ICD-9-CM). The primary diagnosis was stroke, which was further divided into 3 groups: Hemorrhagic stroke (ICD-9 codes 430.0, 431.0, 432.0), ischemic stroke (ICD-9 codes 434.01, 434.11, 434.91, 433.01, 433.11, 433.21, 433.31, 433.81, 433.91, 434.01, 434.11, 434.91, 436), and transient ischemic attacks (TIAs) (ICD-9 codes 435.0, 435.1, 435.2, 435.3, 435.8, 435.9), all of which have been utilized in previous studies^19–21^. DM as a secondary diagnosis was identified using the diagnostic code (250.x). The primary outcome for this study was in-hospital mortality. Secondary outcomes included hospitalization for incident cases of stroke in patients with concomitant diabetes, length of stay (LoS), hospital charges and patient disposition. All patients less than 18 years of age, or with missing age, gender, in-hospital mortality status, length of stay, and total charges were excluded.

### Statistical analysis

Data weighting was used to allow for representative nationwide population estimates as recommended by the AHRQ^18^. Patient-level discharge trend weights consisted of applying the DISCWT variable prior to 2012 and the TRENDWT variable from 2012 to 2014. Patient demographics are presented using frequency distributions and percentages for categorical variable and means (standard deviation) or median (interquartile range) for continuous variables, depending on the distribution. Hospitalizations for incident cases of stroke patients with diabetes are presented per 100,000 individuals using the US population size that is annually reported by the US census bureau (https://www.census.gov). In-hospital mortality is given as age-adjusted and gender-stratified. Spearman correlation was used to assess temporal changes in outcomes. Multivariable logistic regression analysis was conducted to assess the association of in-hospital mortality with clinical characteristics. The model included age, gender, past medical history, cardiovascular risk factors, hospital characteristics and Charlson’s score, a categorized prognostic index that includes 22 different comorbidities^22^. Total hospital charges were adjusted for inflation according to the US bureau of labor statistics (https://data.bls.gov/cgi-bin/cpicalc.pl). Data were analyzed using SPSS (IBM, version 26). A p value < 0.05 was considered statistically significant.

### Ethical approval

The study went through an administrative review only since it did not meet the definition of research involving human subjects given the nature of de-identified data per our institutional review board (IRB), determination letter number 18-00017.

## Results

### Demographics and clinical characteristics of stroke patients with diabetes

**Figure 1:**
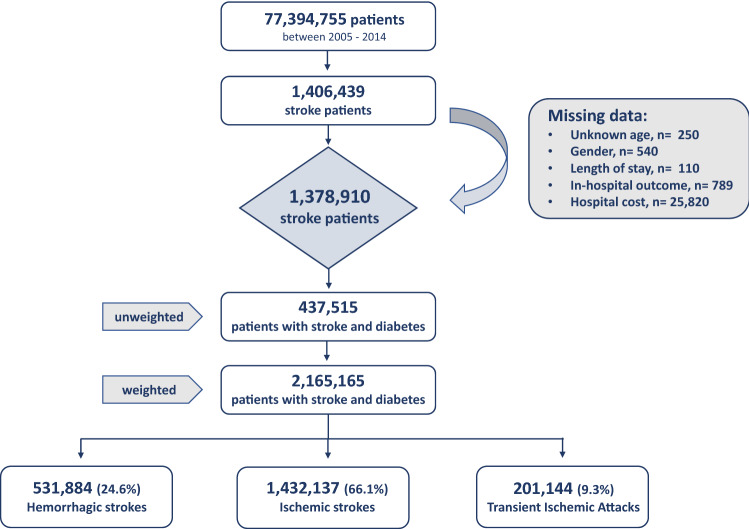
Flow chart of the study.

A total of 1,378,910 (weighted n = 6,822,816) patients between 2005 and 2014 were hospitalized with the primary diagnosis of stroke after exclusion of patients with missing data (Fig. 1). Diabetes as a secondary diagnosis was present in 31.7% of all stroke patients. After weighting, our study sample consisted of 2,165,165 stroke patients with diabetes: 66.1% with ischemic stroke, 24.6% hemorrhagic stroke and 9.3% with TIAs.

The prevalence of diabetes among all stroke patients gradually increased from 28.1% to 35.5% between 2005 and 2014 (p-trend < 0.001) (Supplementary Table 1). Over the 10-year period, the mean (SD) age of stroke patients with diabetes slightly decreased from 70 (13) to 69 (13) years (p-trend < 0.001) (Table 1). Although there were more females than males overall, the proportion of males increased with time (p < 0.001).

**Table 1:** Baseline demographics of diabetic patients admitted for stroke between 2005 to 2014.

Years	2005	2006	2007	2008	2009	2010	2011	2012	2013	2014	P-trend
Total cases	37,828 (28.2)	39,740 (28.8)	39,853 (30.1)	43,593 (30.7)	42,084 (31.4)	44,892 (31.8)	48,799 (32.8)	46,416 (33.9)	46,531 (34.6)	47,779 (35.5)	
Weighted cases	184,759 (8.5)	194,441 (9.0)	196,702 (9.1)	213,372 (9.9)	213,045 (9.8)	224,892 (10.4)	234,325 (10.8)	232,080 (10.7)	232,655 (10.7)	238,895 (11.0)	
Stroke/100 000	17.4	18.1	17.9	19.4	18.6	19.6	21.1	19.8	19.6	20	< 0.001
**Age**											
Mean (SD)	70 (13)	69 (13)	69 (13)	69 (13)	70 (13)	69 (13)	70 (13)	69 (13)	69 (13)	69 (13)	< 0.001
< 55	24,860 (13.5)	27,063 (13.9)	28,086 (14.3)	30,192 (14.1)	29,112 (13.7)	32,582 (14.5)	32,819 (14.0)	32,055 (13.8)	32,945 (14.2)	32,105 (13.4)	0.002
55–64	35,749 (19.5)	40,001 (20.6)	40,461 (20.6)	44,231 (20.7)	44,470 (20.9)	48,193 (21.4)	49,971 (21.3)	50,480 (21.8)	49,690 (21.4)	51,865 (21.7)	< 0.001
65—74	47,875 (25.9)	49,720 (25.6)	49,606 (25.2)	54,260 (25.4)	55,363 (26.0)	58,057 (25.8)	60,331 (25.7)	61,210 (26.4)	63,015 (27.1)	65,415 (27.4)	< 0.001
75–84	54,120 (29.2)	54,284 (27.8)	54,438 (27.6)	58,179 (27.3)	57,554 (27.0)	57,310 (25.5)	60,827 (26.0)	58,440 (25.2)	57,035 (24.5)	59,415 (24.9)	0.083
> 84	22,153 (11.9)	23,373 (12.0)	24,111 (12.2)	26,511 (12.4)	26,545 (12.5)	28,749 (12.8)	30,377 (13.0)	29,895 (12.9)	29,970 (12.9)	30,095 (12.6)	< 0.001
**Gender**											
Male	85,500 (46.3)	90,094 (46.3)	91,738 (46.6)	100,670 (47.2)	100,595 (47.2)	107,976 (48.0)	112,536 (48.0)	112,560 (48.5)	114,575 (49.2)	118,930 (49.8)	< 0.001
**Race**											
White	89,419 (66.1)	90,685 (62.0)	88,112 (60.6)	110,729 (64.0)	114,668 (62.5)	121,782 (60.6)	131,347 (61.6)	138,430 (62.4)	138,775 (62.6)	143,125 (62.5)	< 0.001
Black	22,978 (17.0)	29,094 (19.9)	30,832 (21.2)	34,001 (19.7)	34,639 (18.9)	45,287 (22.5)	45,893 (21.5)	45,000 (20.3)	44,845 (20.2)	46,480 (20.3)	< 0.001
Hispanic	15,600 (11.5)	17,899 (12.2)	16,581 (11.4)	16,500 (9.5)	20,723 (11.3)	21,481 (10.7)	22,598 (10.6)	23,060 (10.4)	23,750 (10.7)	24,080 (10.5)	< 0.001
Asian	3272 (2.4)	3832 (2.6)	4453 (3.1)	4774 (2.8)	5697 (3.1)	5804 (2.90)	5545 (2.6)	6220 (2.8)	6600 (3.0)	7075 (3.1)	< 0.001
Native American	678 (0.5)	1163 (0.8)	1235 (0.8)	1109 (0.6)	1100 (0.6)	1548 (0.80)	1336 (0.6)	1550 (0.7)	1290 (0.6)	1430 (0.6)	0.014
Other	3310 (2.4%)	3653 (2.5)	4136 (2.8)	5797 (3.4)	6553 (3.6)	4955 (2.5)	6471 (3.0)	7605 (3.4)	6515 (2.9)	6990 (3.1)	0.001
**Income**											
Low	57,068 (31.6)	63,842 (33.6)	65,386 (34.1)	68,238 (32.6)	66,225 (31.9)	73,584 (33.5)	75,792 (33.0)	78,750 (34.7)	76,015 (33.4)	78,445 (33.5)	< 0.001
Low-Mid	48,172 (26.7)	49,256 (26.0)	50,370 (26.3)	58,992 (28.2)	56,653 (27.3)	58,047 (26.5)	57,984 (25.2)	58,270 (25.7)	61,365 (26.9)	66,620 (28.5)	< 0.001
High-Mid	42,328 (23.4)	42,027 (22.1)	42,517 (22.2)	45,332 (21.7)	47,725 (23.0)	50,040 (22.8)	56,596 (24.6)	51,205 (22.5)	52,390 (23.0)	51,050 (21.8)	0.01
High	33,039 (18.3)	34,630 (18.2)	33,482 (17.5)	36,614 (17.5)	37,160 (17.9)	37,775 (17.2)	39,532 (17.2)	38,900 (17.1)	37,935 (16.7)	38,035 (16.2)	0.015
**PEP**											
Medicare	122,660 (68.6)	129,939 (66.9)	128,614 (65.5)	137,865 (64.7)	138,398 (65.1)	144,626 (64.5)	154,260 (66.0)	154,330 (66.6)	154,195 (66.4)	157,925 (66.2)	< 0.001
Medicaid	13,179 (7.1)	15,285 (7.9)	14,957 (7.6)	16,410 (7.7)	17,166 (8.1)	19,740 (8.8)	19,511 (8.4)	19,730 (8.5)	19,200 (8.3)	23,430 (9.8)	< 0.001
Private Insurance	33,921 (18.4)	36,241 (18.7)	38,889 (19.8)	43,765 (20.5)	42,120 (19.8)	42,492 (18.9)	42,286 (18.1)	39,270 (17.0)	39,845 (17.1)	41,755 (17.5)	0.162
Self-Pay	7000 (3.8)	7662 (3.9)	8380 (4.3)	9179 (4.3)	9532 (4.5)	11,260 (5.0)	10,877 (4.7)	11,705 (5.1)	11,795 (5.1)	9515 (4.0)	0.006
No charge	860 (0.5)	799 (0.4)	950 (0.5)	963 (0.5)	940 (0.4)	1091 (0.5)	1280 (0.5)	880 (0.4)	1320 (0.6)	915 (0.4)	0.186
Other	3019 (1.6)	4271 (2.2)	4620 (2.4)	4818 (2.3)	4509 (2.1)	5159 (2.3)	5397 (2.3)	5640 (2.4)	6000 (2.6)	5010 (2.1)	0.011
**Comorbidities**											
Obesity	14,540 (7.9)	16,638 (8.6)	19,559 (9.9)	24,646 (11.6)	26,634 (12.5)	29,745 (13.2)	33,157 (14.2)	36,230 (15.6)	39,830 (17.1)	41,230 (17.3)	< 0.001
HTN	148,944 (80.6)	161,541 (83.1)	167,260 (85.0)	183,545 (86.0)	184,997 (86.8)	197,332 (87.7)	208,538 (89.0)	208,415 (89.8)	210,000 (90.3)	216,850 (90.8)	< 0.001
Smoking	23,554 (12.7)	27,623 (14.2)	30,767 (15.6)	36,919 (17.3)	42,131 (19.8)	48,863 (21.7)	55,481 (23.7)	59,115 (25.5)	62,795 (27.0)	73,055 (30.6)	< 0.001
Dyslipidemia	71,253 (38.6)	83,253 (42.8)	91,336 (46.4)	105,232 (49.3)	115,263 (54.1)	127,951 (56.9)	139,990 (59.7)	145,475 (62.7)	149,415 (64.2)	157,545 (65.9)	< 0.001
**Past Medical History**											
Peripheral Vascular Disease	14,653 (7.9)	16,134 (8.3)	17,438 (8.9)	19,707 (9.2)	20,637 (9.7)	21,160 (9.4)	23,813 (10.2)	23,025 (9.9)	23,125 (9.9)	24,445 (10.2)	< 0.001
Valvular Heart Disease	10,130 (5.5)	11,480 (5.9)	15,986 (8.1)	16,464 (7.7)	16,255 (7.6)	16,864 (7.5)	18,817 (8.0)	198,445 (7.9)	19,155 (8.2)	20,300 (8.5)	< 0.001
Renal Failure	17,187 (9.3)	27,012 (13.9)	29,889 (15.2)	34,339 (16.1)	37,586 (17.6)	40,474 (18.0)	45,924 (19.6)	45,125 (19.4)	47,125 (20.3)	49,685 (20.8)	< 0.001
Coronary Artery Disease	51,290 (27.8)	54,330 (27.9)	56,765 (28.9)	63,584 (29.8)	66,714 (31.3)	68,447 (30.4)	73,408 (31.3)	73,445 (31.6)	72,325 (31.1)	73,540 (30.8)	< 0.001
**Hospital Bedsize**											
Small	20,041 (10.8)	234,630 (12.7)	21,856 (11.1)	22,842 (10.7)	21,544 (10.3)	23,391 (10.5)	24,669 (10.7)	26,365 (11.4)	25,785 (11.1)	36,630 (15.3)	0.022
Medium	45,632 (24.7)	48,325 (24.9)	49,796 (25.4)	48,231 (22.6)	50,971 (24.4)	48,310 (21.8)	53,589 (23.2)	59,995 (25.9)	60,615 (26.1)	70,790 (29.6)	0.001
Large	119,085 (64.5)	120,948 (62.4)	124,654 (63.5)	141,947 (66.6)	136,273 (65.3)	150,280 (67.7)	153,083 (66.2)	145,720 (62.8)	146,255 (62.9)	131,475 (55.0)	0.067
**Hospital Location**											
Rural	29,339 (15.9)	28,114 (14.5)	27,391 (14.0)	27,670 (13.0)	25,822 (12.4)	27,612 (12.4)	279,641 (11.9)	24,755 (10.7)	24,130 (10.4)	20,410 (8.5)	0.006
Urban	155,420 (84.1)	165,789 (85.5)	168,915 (86.0)	185,350 (87.0)	182,965 (87.6)	194,369 (87.6)	203,701 (88.1)	207,325 (89.3)	208,525 (89.6)	218,485 (91.5)	< 0.001
**Hospital Region**											
Northeast	34,989 (18.9)	38,003 (19.5)	36,318 (18.5)	37,847 (17.7)	39,865 (18.7)	43,024 (19.1)	42,985 (18.3)	42,135 (18.2)	41,195 (17.7)	43,645 (18.3)	0.001
Midwest	43,273 (23.4)	44,979 (23.1)	45,205 (23.0)	47,646 (22.3)	46,098 (21.6)	49,268 (21.9)	53,278 (22.7)	48,900 (21.1)	49,155 (21.1)	49,430 (20.7)	0.028
South	79,081 (42.8)	81,118 (41.7)	83,880 (42.6)	97,022 (45.5)	90,785 (42.6)	96,734 (43.0)	99,210 (42.3)	103,495 (44.6)	104,175 (44.8)	106,330 (44.5)	< 0.001
West	27,416 (14.8)	30,342 (15.6)	31,299 (15.9)	30,857 (14.5)	36,297 (17.0)	35,866 (15.9)	38,851 (16.6)	37,550 (16.2)	38,130 (16.4)	39,490 (16.5)	0.001
**Charlson’s Score**											
0	3603 (2.0)	3839 (2.0)	4465 (2.3)	4082 (1.9)	3512 (1.6)	3687 (1.6)	3710 (1.6)	3535 (1.5)	3690 (1.6)	4240 (1.8)	0.888
1	77,745 (42.1)	83,215 (42.8)	80,346 (40.8)	76,032 (35.6)	73,410 (34.5)	75,854 (33.7)	76,461 (32.6)	73,940 (31.9)	73,515 (31.6)	73,650 (30.8)	0.012
2	45,207 (24.5)	47,798 (24.6)	48,171 (24.5)	54,167 (25.4)	52,430 (24.6)	53,939 (24.0)	56,258 (24.0)	55,200 (23.8)	54,170 (23.3)	53,870 (22.5)	0.004
> = 3	58,203 (31.5)	59,589 (30.6)	63,721 (32.4)	79,092 (37.1)	83,693 (39.3)	91,412 (40.6)	97,896 (41.8)	99,405 (42.8)	101,280 (43.5)	107,135 (44.8)	< 0.001

### Cardiovascular outcomes

**Figure 2:**
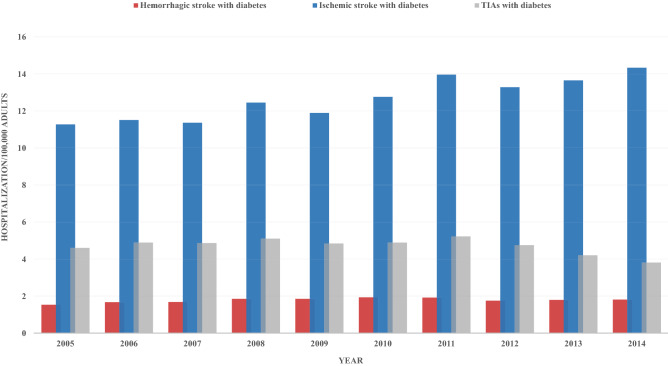
Trends in hospitalizations per 100,000 US adults for incident cases.

Hospitalizations for incident cases of stroke and diabetes followed a distinct trend. For both hemorrhagic and ischemic stroke groups, incident cases significantly increased from 1.5 to 1.8 / 100,000 US adults (p-trend < 0.001) and 11.3 to 14.3 / 100,000 US adults, respectively (p-trend < 0.001 for both). However, incident cases of TIAs had a significant decrease over the same 10-year period, dropping from 4.6 to 3.8 / 100,000 US adults (p-trend < 0.001) (Fig. 2).

**Figure 3:**
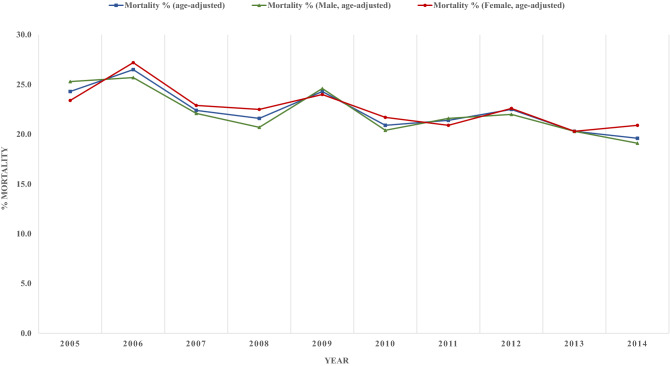
In hospital mortality among patients with diabetes admitted for hemorrhagic stroke.

The age-ajusted in-hospital mortality amongst patients admitted for hemorrhagic stroke showed a decrease by almost 5%, from 24.3% to 19.6% (p-trend < 0.001). Similarly, the gender stratified age-adjusted in-hospital mortality decreased in both males and females, from 25.3% to 19.1% (p-trend = 0.004) and 23.4% to 20.9% (p = 0.007), respectively (Fig. 3).

**Figure 4:**
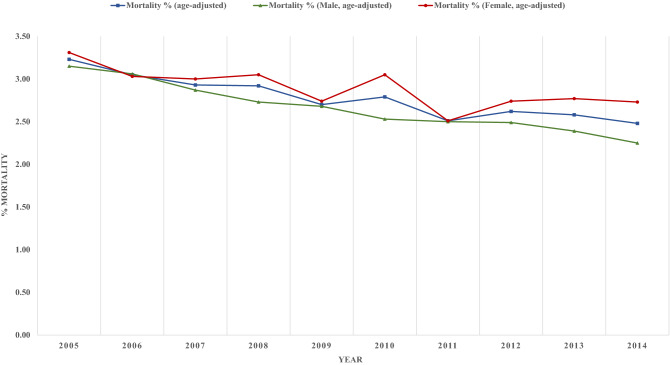
In hospital mortality among patients with diabetes admitted for ischemic stroke.

Age-adjusted in-hospital mortality decreased from 3.23% to 2.48% in patients with ischemic stroke (p-trend < 0.001) (Fig. 4). Further, age-adjusted mortality significantly decreased in males from 3.15% to 2.25% (p-trend = 0.025) and in females, dropping from 3.31% to 2.73% (p-trend = 0.002).

In-hospital mortality in patients hospitalized for TIAs was low (crude mortality 0.1%, adjusted mortality 0.073% in 2005) and remained almost unchanged during the course of follow-up (crude mortality 0.2%, adjusted mortality 0.098% in 2014). No clear pattern for gender distribution was found.

Furthermore, we examined other predictors of mortality among the 3 stroke sub-groups. As expected, older patients had a higher risk of stroke. For instance, the risk of TIAs was 5-times higher in patients > 85 years old compared to patients < 55 years old. Women were more predisposed to hemorrhagic stroke. Interestingly, African Americans and Latinos with diabetes had a 20% average lower risk of developing a stroke, compared to their Caucasian counterparts. A history of cardiovascular diseases such as renal failure, CAD, peripheral vascular disease and a Charlson index > 3 also contributed to an increased stroke risk. Unexpectedly, DM patients with cardiovascular risk factors such as obesity, hypertension, smoking and dyslipidemia had significantly lower odds of in-hospital mortality compared to diabetic patients without these comorbidities (Table 2).

**Table 2:** Predictors of mortality in patients with diabetes admitted for stroke per stroke type.

	Hemorrhagic Stroke		Ischemic Stroke		TIAs	
	Adjusted OR (95% CI)	p-value	Adjusted OR (95% CI)	p-value	Adjusted OR (95% CI)	p-value
**Age**						
< 55 (ref)	Ref	Ref	Ref	Ref	Ref	Ref
55–64	1.12 (1.08–1.16)	< 0.001	1.15 (1.10–1.20)	< 0.001	0.89 (0.57–1.37)	0.59
65–74	1.14 (1.09–1.19)	< 0.001	1.76 (1.69–1.84)	< 0.001	1.88 (1.25–2.83)	0.003
75–84	1.33 (1.28–1.39)	< 0.001	2.55 (2.44–2.66)	< 0.001	3.31 (2.22–4.95)	< 0.001
> 85	1.35 (1.29–1.42)	< 0.001	3.70 (3.54–3.87)	< 0.001	5.01 (3.32–7.57)	< 0.001
**Gender**						
Male (Ref*)	Ref	Ref	Ref	Ref	Ref	Ref
Female	1.05 (1.02–1.07)	< 0.001	1.00 (0.99–1.02)	0.75	0.86 (0.74–1.01)	0.064
**Race**						
White (Ref)	Ref	Ref	Ref	Ref	Ref	Ref
Black	0.87 (0.84–0.90)	< 0.001	0.75 (0.73–0.77)	< 0.001	0.83 (0.66–1.02)	0.081
Hispanic	0.81 (0.78–0.84)	< 0.001	0.88 (0.85–0.91)	< 0.001	0.63 (0.47–0.86)	0.003
Asian	0.96 (0.91–1.01)	0.15	0.90 (0.86–0.96)	< 0.001	0.61 (0.32–1.17)	0.13
Native American	0.89 (0.77–1.02)	0.1	1.00 (0.89–1.12)	0.97	1.21 (0.5–2.94)	0.67
Other	1.08 (1.02–1.14)	0.007	1.11 (1.05–1.17)	< 0.001	0.55 (0.29–1.03)	0.061
**Income**						
Low (Ref)	Ref	Ref	Ref	Ref	Ref	Ref
Low-Mid	1.03 (0.97–1.10)	0.27	0.99 (0.96–1.01)	0.21	0.81 (0.66–0.99)	0.044
High-Mid	0.94 (0.89–1.00)	0.09	0.95 (0.93–0.98)	< 0.001	0.54 (0.42–0.68)	< 0.001
High	1.06 (0.99–1.13)	0.11	0.96 (0.93–0.99)	0.003	1.08 (0.88–1.33)	0.45
**Primary Expected Payer**						
Medicare (Ref)	Ref	Ref	Ref	Ref	Ref	Ref
Medicaid	0.87 (0.83–0.91)	< 0.001	1.41 (1.35–1.47)	< 0.001	1.93 (1.24–2.69)	0.002
Private Insurance	0.86 (0.83–0.89)	< 0.001	1.21 (1.18–1.25)	< 0.001	0.95 (0.70–1.27)	0.71
Self-Pay	1.20 (1.13–1.27)	< 0.001	1.59 (1.50–1.67)	< 0.001	1.29 (0.67–2.49)	0.44
No Charge	0.62 (0.51–0.74)	< 0.001	1.19 (1.01–1.40)	0.039	0.00 (0.00–0.00)	0.99
Other	1.15 (1.07–1.23)	< 0.001	2.37 (2.25–2.50)	< 0.001	0.75 (0.30–1.85)	0.53
**Obesity**						
No (Ref)	Ref	Ref	Ref	Ref	Ref	Ref
Yes	0.84 (0.81–0.87)	< 0.001	0.88 (0.86–0.91)	< 0.001	0.85 (0.64–1.13)	0.27
**Hypertension**						
No (Ref)	Ref	Ref	Ref	Ref	Ref	Ref
Yes	0.87 (0.84–0.90)	< 0.001	0.70 (0.69–0.72)	< 0.001	0.81 (0.66–0.99)	0.04
**Smoking**						
No (Ref)	Ref	Ref	Ref	Ref	Ref	Ref
Yes	0.78 (0.76–0.81)	< 0.001	0.73 (0.71–0.75)	< 0.001	0.72 (0.57–0.91)	0.006
Dyslipidemia						
No (Ref)	Ref	Ref	Ref	Ref	Ref	Ref
Yes	0.61 (0.60–0.63)	< 0.001	0.52 (0.51–0.53)	< 0.001	0.52 (0.45–0.61)	< 0.001
**Peripheral vascular disease**						
No (Ref)	Ref	Ref	Ref	Ref	Ref	Ref
Yes	–	–	1.09 (1.06–1.13)	< 0.001	1.19 (0.95–1.48)	0.12
**Valvular Heart Disease**						
No (Ref)	Ref	Ref	Ref	Ref	Ref	Ref
Yes	0.80 (0.76–0.84)	< 0.001	0.86 (0.84–0.89)	< 0.001	0.99 (0.76–1.30)	0.95
**Renal Failure**						
No (Ref)	Ref	Ref	Ref	Ref	Ref	Ref
Yes	1.28 (1.24–1.32)	< 0.001	1.38 (1.35–1.41)	< 0.001	2.10 (1.78–2.48)	< 0.001
**CAD**						
No (Ref)	Ref	Ref	Ref	Ref	Ref	Ref
Yes	1.27 (1.24–1.31)	< 0.001	1.05 (1.03–1.07)	< 0.001	0.94 (0.80–1.10)	0.42
**Hospital Bedsize**						
Small (Ref)	Ref	Ref	Ref	Ref	Ref	Ref
Medium	–	–	1.08 (1.05–1.12)	< 0.001	1.73 (1.32–2.28)	< 0.001
Large	–	–	1.23 (1.19–1.26)	< 0.001	1.35 (1.04–1.74)	0.025
**Hospital Location**						
Rural (Ref)	Ref	Ref	Ref	Ref	Ref	Ref
Urban	0.80 (0.76–0.84)	< 0.001	1.03 (1.00–1.05)	0.099	–	–
**Hospital Region**						
Northeast (Ref)	Ref	Ref	Ref	Ref	Ref	Ref
Midwest	0.91 (0.87–0.94)	< 0.001	0.88 (0.85–0.90)	< 0.001	0.67 (0.52–0.86)	0.002
South	0.95 (0.92–0.98)	< 0.001	0.90 (0.88–0.92)	< 0.001	0.89 (0.74–1.07)	0.22
West	0.98 (0.95–1.02)	0.39	0.96 (0.93–0.99)	0.005	0.84 (0.65–1.08)	0.18
**Charlson's Score**						
0 (Ref)	Ref	Ref	Ref	Ref	Ref	Ref
1	1.93 (1.74–2.14)	< 0.001	0.91 (0.84–0.99)	0.034	0.88 (0.47–1.66)	0.69
2	1.84 (1.66–2.04)	< 0.001	1.12 (1.03–1.22)	0.009	2.06 (0.56–2.00)	0.87
> = 3	1.34 (1.21–1.49)	< 0.001	1.65 (1.51–1.79)	< 0.001	2.11 (1.12–3.97)	0.02

### Socio-economic outcomes

**Figure 5:**
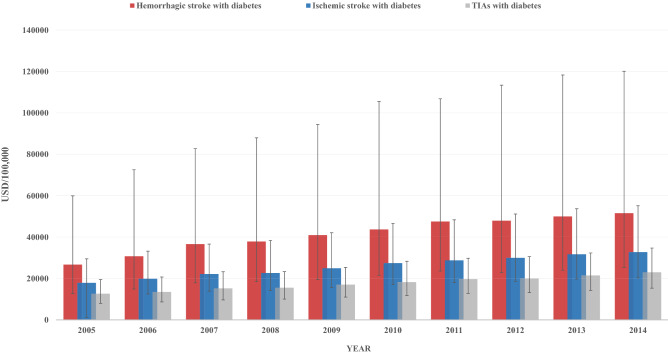
Median (IQR) total hospital charges (USD/stay—adjusted for inflation) of patients with diabetes and hemorrhagic stroke, ischemic stroke, and TIAs.

After adjusting for inflation, the total charges per hospital stay had a general two-fold increasing trend in all stroke groups. Median (IQR) hospital charges increased from USD 26684 (12701-59919) to USD 51538 (25345-120105) in the hemorrhagic stroke group, USD 17886 (1174-29482) to USD 32723 (20290-55128) in the ischemic stroke group, and USD 12644 (7975-19469) to USD 22993 (15331-34692) in the TIAs group (p-trend < 0.001 for all) (Fig. 5).

Between 2005 and 2014, in hospital LoS had a slight but statistically significant decrease. In all stroke patients with diabetes, median (IQR) LoS decreased from 4 (2–6) to 3 (2–6) days (p-trend < 0.001). However, the trend was not homogenous within the 3 groups: LoS moderately increased from 5 (2–10) to 5 (2–11) days (p-trend = 0.032) in the hemorrhagic stroke group but decreased in the ischemic stroke group from 4 (3–7) in 2005 to 4 (2–6) days in 2014; p-trend < 0.001 and in the TIAs group from 3 (2–4) days in 2005 to 2 (1–3) days in 2014; p-trend < 0.001. (Supplementary Table 2).

In patients with hemorrhagic stroke, there was an upward trend of discharge to a long-term facility by almost 5% from 2005 till 2010 (Supplementary table 3). Interestingly, disposition to a long-term facility slightly but significantly decreased from 44.9% in 2010 to 44.5% in 2014, which was counteracted by a non-significant increase in short term facilities and a steady significant one to home discharge. In TIAs, the vast majority of patients were discharged home (data not shown).

## Discussion

Our retrospective analysis over a 10-year period provides a general outlook on mortality and socio-economic trends in DM patients admitted for stroke in the US. We have shown that the prevalence of diabetes as well as hospitalizations for incident cases of stroke patients with diabetes have increased while age-adjusted mortality has continued to decline. These changes occurred in the setting of doubling in healthcare costs despite adjustment for inflation.

There has been an overall decrease in the stroke incidence and mortality amongst the general population, which seems applicable to patients with diabetes as shown in our study. This decrease has been observed worldwide in both high and low-income countries^23^. In the US, while the age-adjusted incidence of stroke is decreasing*,* its prevalence continues to rise^24^. This is the result on an aging of population that drives higher the lifetime risk of developing a stroke, coupled with an overall decrease in age-adjusted stroke-related mortality^3,25,26^.

In the Tromso study, up to 60% of the temporal reduction in stroke incidence over a 17-year follow up period was attributed to the modification of cardiovascular risk factors^27^. With advancements in diagnostic and therapeutic strategies for stroke, as well as the implementation of primary and secondary prevention measures, further decreases in incidence and mortality are expected, which may be counterbalanced by an increased stroke prevalence in the general population.

Pre-diabetes and diabetes are major risk factors for stroke^5^. The risk is almost doubled in both genders with women experiencing a higher risk and worse prognosis^8^. Nevertheless, a decrease in cause-specific mortality of patients with cardiovascular disease has been documented during the last two decades^16^. Using the same NIS database, we have recently reported a steady decline in age-adjusted mortality in heart failure and diabetes between 2005 and 2014^28^. A similar observation was reported in diabetic patients hospitalized for acute myocardial infarction during the same period of time^17^. Our results appear concordant with several other studies reporting a decrease in mortality in patients with diabetes and cardiovascular disease in the US.

The classification of TIAs is challenging. TIAs were initially described using a “time-based” definition as focal neurological signs or symptoms that resolve in less than 24 h^29^. This was consequently followed by a shift towards a tissue-based definition, which describes a TIAs as a transient neurological event without clinical or radiographic evidence of an acute infarction^29^. Studies have suggested that redefining TIAs will account for better prognosis in both TIAs and ischemic strokes, and reduce the annual incidence of TIAs^30,31^. This correlates with the trend analysis of our study that illustrates a statistically significant decrease in TIAs-related hospitalizations over the chosen 10-year time period. Furthermore, patients presenting to the hospital with a suspected TIA tend to get managed and discharged directly from the emergency room as stroke management guidelines evolve over time. This means that a large number of TIAs patients presenting to the hospital do not get admitted as inpatients, thus further justifying the decreasing trend in TIAs cases. Similarly, many patients fail to report to a health care facility after experiencing transient neurological symptoms, further contributing to the decreasing trend^32^.

The overall decrease in stroke-related in-hospital mortality, reported in several studies in the general patient population, comes at a price of an increase in the economic burden of stroke^25^. Cardiovascular disease, all pathologies included, is the leading cause of medical expenditure in the US, according to the latest heart disease and stroke statistics^2^. Stroke-related healthcare costs were estimated to be around 45.5 billion USD, and appear to follow a yearly ascending trend^2^. The cost of diabetes with its risk factors and cardiovascular complications was estimated to be 237 billion USD in 2017, which represents a 26% increase over 5 years^33^.

Our results are aligned with several studies that assessed the economic burden of diabetes on cardiovascular complications. The presence of CVD in patients with diabetes has been shown to significantly increase care costs^34^. In a systematic review, Einarso et al. estimated that the presence of stroke in patients with DM resulted in a threefold increase in costs as compared to patients with diabetes without Stroke^35^. We reported an almost two-fold increase in the total cost per hospital stay over the defined 10-year period despite a slight decrease in LoS among stroke patients. This is congruent with the forecasted threefold increase in costs between 2010 and 2030 in the US^36^.

In our study, an unexpected relationship emerged between several atherosclerotic risk factors and in-hospital mortality, including smoking, hypertension, dyslipidemia and obesity. Ahmed et al. also reported in a recent analysis of the NIS that smokers and obese patients had a lower in-hospital mortality in myocardial infarction patients with diabetes^17^. These findings may be explained by several factors. First, there has been an increase in the screening and documentation of these comorbidities, especially as they pertain to patients with cardiovascular and cerebrovascular disease. It is reasonable to assume that increased identification of these modifiable risk factors has resulted in more targeted risk-factor modifications, thus influencing outcomes. Moreover, primary and secondary prevention strategies, including medications and lifestyle modifications have been more aggressively advocated and implemented. Lastly, pharmacological agents used in risk-factor modification, such as statins, ACE-inhibitors and ARBs, may convey benefit through multiple mechanisms, such as cardioprotective and anti-inflammatory effects. These effect-combinations may further reduce mortality due to stroke and other cardiovascular diseases.

One of the limitations of our study is the absence of hospital-level data in the NIS database, including patients’ medications at the time of admission or during their hospital stay. Other important parameters, such as HBA_1C_, duration of DM and blood pressure, were also not available. Additionally, it was not possible to differentiate type 1 and type 2 diabetes since most patients in the NIS were simply labelled as diabetics. These factors act as potentially major confounding variables in assessing predictors of stroke-related in-hospital mortality.

A second limitation involves TIAs patients given the relatively subjective nature of the diagnosis and the constantly evolving definition, which may result in a level of reporting error of primary diagnoses as TIAs *versus* ischemic stroke or other diagnosis. On the other hand, this study provides trend analysis and outcome-data that has been collected under strict and specific standards across different hospitals in the US. The data were collected over 10 years and is fairly representative of the US population after weighting, thus providing a very large sample for analysis.

In total, despite the increasing prevalence of diabetes and its associated cardiovascular risk factors and complications over the past decade, in-hospital mortality for stroke patients with diabetes is on the decline. This has occurred in parallel with a doubling in stroke-related hospitalization costs. In the future, we expected that widespread risk factor screening, coupled with aggressive primary and secondary prevention measures, will continue to reduce the burden of stroke, thus decreasing the burden of stroke-related disability in diabetic patients.

## Supplementary Information

Supplementary Information

## Data Availability

The NIS is a publicly available database.
