# Supplementary material for: Mortality and socio-economic outcomes among patients hospitalized for stroke and diabetes in the US: a recent analysis from the National Inpatient Sample
Source: Sci Rep. 2021 Apr 15;11:8204. doi: 10.1038/s41598-021-87320-w (PMC8050299; doi:10.1038/s41598-021-87320-w)
Supplement: Supplementary file 1 — Supplementary Information [file 41598_2021_87320_MOESM1_ESM.docx]

**Mortality and socio-economic outcomes among patients hospitalized for stroke and diabetes in the US: A recent analysis from the National Inpatient Sample**

Aya Tabbalat, MD **^1^**, Soha Dargham, MSc **^1^**, Jassim Al Suwaidi, MBChB **^1,2^**, Samar Aboulsoud, MBChB ^3^, Salman Al Jerdi, MD ^1^, Charbel Abi Khalil, MD, PhD **^1,2,4*^**

1. Research department. Weill Cornell Medicine-Qatar. Doha-Qatar.
2. Heart Hospital. Hamad Medical Corporation. Doha-Qatar.
3. Department of Medicine. Kasr Alainy. Cairo university. Egypt, Cairo
4. Joan and Sanford I. Weill Department of Medicine. Weill Cornell Medicine. New York, US

*** Correspondent author:** Charbel Abi Khalil. Weill Cornell Medicine-Qatar. PO box 24144. Doha-Qatar. Tel: +97444928284. Email: [cha2022@med.cornell.edu](mailto:cha2022@med.cornell.edu)

**Supplementary tables**

**Supplementary Table 1:** Baseline characteristics of all patients with stroke in the NIS database.

| Years | 2005 | 2006 | 2007 | 2008 | 2009 | 2010 | 2011 | 2012 | 2013 | 2014 | P-trend |
| --- | --- | --- | --- | --- | --- | --- | --- | --- | --- | --- | --- |
| Age |  |  |  |  |  |  |  |  |  |  |  |
| Mean (SD) | 71 | 71 | 71 | 70 | 70 | 70 | 71 | 70 | 70 | 70 | <0.001 |
| <55 | 20702 (15.4%) | 22416 (16.3%) | 21476 (16.2%) | 23575 (16.6%) | 22088 (16.5%) | 23840 (16.9%) | 23890 (16.1%) | 21879 (16.0%) | 21510 (16.0%) | 21366 (15.9%) | 0.002 |
| 55-64 | 20692 (15.4%) | 22513 (16.3%) | 21971 (16.6%) | 24031 (16.9%) | 22890 (17.1%) | 25553 (18.1%) | 26248 (17.7%) | 25077 (18.3%) | 24637 (18.3%) | 25138 (18.7%) | <0.001 |
| 65 - 74 | 27786 (20.7%) | 28232 (20.5%) | 27009 (20.4%) | 29226 (20.6%) | 28247 (21.1%) | 29423 (20.9%) | 31251 (21.0%) | 29861 (21.8%) | 30029 (22.3%) | 30578 (22.7%) | <0.001 |
| 75-84 | 39562 (29.5%) | 39228 (28.5%) | 37034 (28.0%) | 38657 (27.2%) | 35798 (26.7%) | 35611 (25.3%) | 38292 (25.8%) | 34117 (24.9%) | 32760 (24.4%) | 32525 (24.2%) | 0.038 |
| >84 | 25514 (19.0%) | 25382 (18.4%) | 24924 (18.8%) | 26620 (18.7%) | 25136 (18.7%) | 26605 (18.9%) | 28990 (19.5%) | 26140 (19.1%) | 25514 (19.0%) | 24848 (18.5%) | <0.001 |
| Gender |  |  |  |  |  |  |  |  |  |  |  |
| Male | 59145 (44.1%) | 61411 (44.6%) | 59071 (44.6%) | 63746 (44.9%) | 60937 (45.4%) | 64731 (45.9%) | 67817 (45.6%) | 63313 (46.2%) | 63420 (47.2%) | 64134 (47.7%) | <0.001 |
| Race |  |  |  |  |  |  |  |  |  |  |  |
| White | 73893 (74.8%) | 72496 (71.2%) | 66866 (69.5%) | 83028 (72.1%) | 80612 (70.6%) | 86710 (69.2%) | 94569 (70.2%) | 91589 (70.3%) | 89421 (70.2%) | 89612 (69.9%) | <0.001 |
| Black | 12607 (12.8%) | 15333 (15.1%) | 15526 (16.1%) | 17173 (14.9%) | 16932 (14.8%) | 22146 (17.7%) | 22425 (16.7%) | 20561 (15.8%) | 20475 (16.1%) | 20711 (16.2 %) | <0.001 |
| Hispanic | 7662 (7.8%) | 8833 (8.7%) | 8231 (8.6%) | 8023 (7.0%) | 9380 (8.2%) | 9570 (7.6%) | 10366 (7.7%) | 10187 (7.8%) | 10307 (8.1%) | 10291 (8.0%) | 0.12 |
| Asian | 2113 (2.1%) | 2443 (2.4%) | 2543 (2.6%) | 2808 (2.4%) | 3111 (2.7%) | 3213 (2.6%) | 2900 (2.2%) | 3228 (2.5%) | 3339 (2.6%) | 3369 (2.6%) | 0.21 |
| Native American | 294 (0.3%) | 544  (0.5 %) | 649  (0.7%) | 596 (0.5 %) | 580 (0.5%) | 765 (0.6%) | 609 (0.5%) | 712 (0.5%) | 521 (0.4%) | 607 (0.5%) | 0.041 |
| Other | 2153 (2.2%) | 2176 (2.1%) | 2359 (2.5%) | 3459 (3.0%) | 3573 (3.1%) | 2832 (2.3%) | 3772 (2.8%) | 4029 (3.1%) | 3393 (2.7%) | 3553 (2.8%) | 0.001 |
| Income |  |  |  |  |  |  |  |  |  |  |  |
| Low | 37144 (28.3%) | 39973 (29.7%) | 39383 (30.5%) | 40992(29.4%) | 37418 (28.6%) | 41566 (30.2%) | 42275 (29.0%) | 41529 (30.9%) | 39290 (30.0%) | 39486 (30.0%) | <0.001 |
| Low-Mid | 33798 (25.7%) | 34804 (25.9%) | 33639(26.0%) | 39096 (28.0%) | 35275 (26.9%) | 36064 (26.2%) | 36105 (24.8%) | 33891 (25.3%) | 35236 (26.8%) | 37042 (28.1%) | <0.001 |
| High-Mid | 32041 (24.4%) | 33639 (26.0%) | 29543 (22.9%) | 30884 (22.2 %) | 30954 (23.6%) | 32424 (23.5%) | 37325 (25.6%) | 31517 (23.5%) | 31183 (23.7%) | 29908 (22.7%) | 0.015 |
| High | 28374 (21.6%) | 28096 (20.9%) | 26588 (20.6%) | 28388 (20.4%) | 27284 (20.8%) | 27700 (20.1%) | 30168 (20.7%) | 27263 (20.3%) | 25844 (19.6%) | 25273  (19.2%) | 0.012 |
| PEP |  |  |  |  |  |  |  |  |  |  |  |
| Medicare | 91146 (67.9%) | 91114 (66.2%) | 85900 (65.0%) | 90582 (63.8%) | 85519 (63.9%) | 88638 (63.0%) | 96851 (65.3%) | 89486 (65.4%) | 87678 (65.3%) | 86829 (64.7%) | <0.001 |
| Medicaid | 7755 (5.8%) | 8476 (6.2%) | 8136 (6.2%) | 9245 (6.5%) | 9125 (6.8%) | 10846 (7.7%) | 10446 (7.0%) | 9883 (7.2%) | 9644 (7.2%) | 12045 (9.0%) | <0.001 |
| Private Insurance | 26824 (20.0%) | 28348 (20.6%) | 28448 (21.5%) | 31565 (22.2%) | 28944 (21.6%) | 29524 (21.0%) | 29364 (19.8%) | 25898 (18.9%) | 25288 (18.8%) | 26179 (19.5%) | 0.621 |
| Self-Pay | 5426 (4.0%) | 5882 (4.3%) | 5932 (4.5%) | 6418 (4.5%) | 6665 (5.0%) | 7498 (5.3%) | 7321 (4.9%) | 7486 (5.5%) | 7353 (5.5%) | 5832 (4.3%) | 0.62 |
| No charge | 656 (0.5%) | 548 (0.4%) | 579 (0.4%) | 721 (0.5%) | 648 (0.5%) | 723 (0.5%) | 719 (0.5%) | 548 (0.4%) | 832 (0.6%) | 550 (0.4%) | 0.56 |
| Other | 2332 (1.7%) | 3212 (2.3%) | 3171 (2.4%) | 3356 (2.4%) | 2989 (2.2%) | 3507 (2.5%) | 3534 (2.4%) | 3429 (2.5%) | 3481 (2.6%) | 2823 (2.1%) | <0.001 |
| Comorbidities |  |  |  |  |  |  |  |  |  |  |  |
| Diabetes | 37828 (28.2%) | 39740 (28.8%) | 39853 (30.1%) | 43593 (30.7%) | 42084 (31.4%) | 44892 (31.8%) | 48799 (32.8%) | 46416 (33.9%) | 46531 (34.6%) | 47779 (35.5%) | <0.001 |
| Obesity | 5971 (4.4%) | 6679 (4.8%) | 7490 (5.7%) | 9580 (6.7%) | 9893 (7.4%) | 10874 (7.7%) | 12607 (8.5%) | 13016 (9.5%) | 14143 (10.5%) | 14684 (10.9%) | <0.001 |
| HTN | 95684 (71.3%) | 100740 (73.1%) | 100141 (75.6%) | 108620 (76.5%) | 104741 (78.1%) | 111031 (78.7%) | 119104 (80.1%) | 111095 (81.0%) | 110070 (81.9%) | 110652 (82.3%) | <0.001 |
| Smoking | 20830 (15.5%) | 23596 (17.1%) | 24261 (18.3%) | 28649 (20.1%) | 29823 (22.2%) | 33993 (24.1%) | 37889 (25.5%) | 37550 (27.3%) | 39093 (28.9%) | 43121 (31.9%) | <0.001 |
| Dyslipidemia | 42269 (31.5%) | 48069 (34.9%) | 50852 (38.4%) | 58719 (41.3%) | 61052 (45.5%) | 67260 (47.7%) | 75650 (50.8%) | 72900 (52.9%) | 73437 (54.4%) | 74482 (55.1%) | <0.001 |
| Past Medical History |  |  |  |  |  |  |  |  |  |  |  |
| Peripheral Vascular Disease | 8738 (6.5%) | 9390 (6.8%) | 9625 (7.3%) | 10979 (7.7%) | 10992 (8.2%) | 11089 (7.9%) | 12676 (8.5%) | 11547 (8.4%) | 11419 (8.5%) | 11799 (8.8%) | <0.001 |
| Valvular Heart Disease | 8849 (6.6%) | 9543 (6.9%) | 12754 (9.6%) | 12456 (8.8%) | 11846 (8.8%) | 12033 (8.5%) | 13610 (9.2%) | 12376 (9.0%) | 12469 (9.3%) | 12517 (9.3%) | <0.001 |
| Renal Failure | 7475 (5.6%) | 12008 (8.7%) | 13077 (9.9%) | 14454 (10.2%) | 15192 (11.3%) | 16602 (11.8%) | 19123 (12.9%) | 17935 (13.1%) | 18206 (13.5%) | 18981 (14.1%) | <0.001 |
| Coronary Artery Disease | 28639 (21.3%) | 29762 (21.6%) | 29179 (22.0%) | 32246 (22.7%) | 31671 (23.6%) | 32362 (22.9%) | 35663 (24.0%) | 32951 (23.9%) | 32057 (23.7%) | 31824 (23.6%) | <0.001 |
| Hospital Bedsize |  |  |  |  |  |  |  |  |  |  |  |
| Small | 14677 (10.9%) | 17993 (13.1%) | 16134 (12.2%) | 15669 (11.0%) | 13902 (10.6%) | 16110 (11.6%) | 16332 (11.1%) | 15649 (11.4%) | 15006 (11.2%) | 20686 (15.4%) | 0.012 |
| Medium | 32873 (24.5%) | 33682 (24.5%) | 32172 (24.3%) | 32930 (23.2%) | 31591 (24.0%) | 30296 (21.8%) | 32858 (22.4%) | 34822 (25.4%) | 34598 (25.7%) | 39209 (29.2%) | <0.001 |
| Large | 86706 (64.6%) | 85726 (62.4%) | 32930 (23.2%) | 93350 (65.8%) | 86174 (65.4%) | 92767 (66.7%) | 97519 (66.5%) | 86603 (63.2%) | 84846 (63.1%) | 74560 (55.5%) | 0.072 |
| Hospital Location |  |  |  |  |  |  |  |  |  |  |  |
| Rural | 20264 (15.1%) | 19393 (14.1%) | 19113 (14.5%) | 18509 (13.0%) | 16351 (12.4%) | 18455 (13.3%) | 15847 (10.8%) | 14758 (10.8%) | 13904 (10.3%) | 11329 (8.4%) | 0.001 |
| Urban | 113992 (84.9%) | 118008 (85.9%) | 113095 (85.5%) | 123395 (87.0%) | 115316 (87.6%) | 120718 (86.7%) | 130862 (89.2%) | 122316 (89.2%) | 120546 (89.7%) | 123126 (91.6%) | <0.001 |
| Hospital Region |  |  |  |  |  |  |  |  |  |  |  |
| Northeast | 25768 (19.2%) | 25424 (18.5%) | 22884 (17.3%) | 27125 (19.1%) | 25219 (18.8%) | 27379 (19.4%) | 27961 (18.8%) | 25969 (18.9%) | 25332 (18.8%) | 25446 (18.9%) | 0.001 |
| Midwest | 31036 (23.1%) | 30966 (22.5%) | 29847 (22.5%) | 31409 (22.1%) | 29750 (22.2%) | 30816 (21.9%) | 34848 (23.4%) | 29553 (21.6%) | 28855 (21.5%) | 29070 (21.6%) | 0.001 |
| South | 55794 (41.6%) | 58591 (42.5%) | 55265 (41.7%) | 62090 (43.7%) | 55350 (41.3%) | 57782 (41.0%) | 60693 (40.8%) | 58059 (42.4%) | 56979 (42.4%) | 56831 (42.3%) | <0.001 |
| West | 21658 (16.1%) | 22790 (16.5%) | 24418 (18.4%) | 21485 (15.1%) | 25055 (17.8%) | 25055 (17.8%) | 25169 (16.9%) | 23493 (17.1%) | 23284 (17.3%) | 23108 (17.2%) | 0.18 |
| Charlson’s Score |  |  |  |  |  |  |  |  |  |  |  |
| 0 | 248038 (37.8%) | 258963 (38.4%) | 239228 (36.6%) | 227094 (32.6%) | 212815 (31.3%) | 213984 (30.3%) | 209601 (29.4%) | 196120 (28.6%) | 187675 (27.9%) | 183415 (27.3%) | <0.001 |
| 1 | 176595 (26.9%) | 183896 (27.2%) | 177293 (27.1%) | 183748 (26.4%) | 177635 (26.1%) | 181302 (25.7%) | 181406 (25.4%) | 172085 (25.1%) | 167245 (24.9%) | 165220 (24.6%) | 0.001 |
| 2 | 125944 (19.2%) | 127046 (18.8%) | 126632 (19.4%) | 146272 (21.0%) | 141036 (20.8%) | 149441 (21.2%) | 149902 (21.0%) | 145395 (21.2%) | 142570 (21.2%) | 140590 (20.9%) | 0.042 |
| >=3 | 105199 (16.0%) | 104949 (15.6%) | 110716 (16.9%) | 139556 (20.0%) | 147832 (21.8%) | 161802 (22.9%) | 172232 (24.2%) | 171770 (25.1%) | 174760 (26.0%) | 183050 (27.2%) | <0.001 |

**Supplementary Table 2:** *Length of stay (LOS) in patients with diabetes admitted for stroke, according to stroke type*

| Years | 2005 | 2006 | 2007 | 2008 | 2009 | 2010 | 2011 | 2012 | 2013 | 2014 | P-value |
| --- | --- | --- | --- | --- | --- | --- | --- | --- | --- | --- | --- |
| LoS in patients with hemorrhagic stroke and DM (IQR) | 5  (2-10) | 6  (3-11) | 6  (3-12) | 6  (3-11) | 5  (3-11) | 6  (3-11) | 5  (3-10) | 5  (3-11) | 5  (3-10) | 5  (2-11) | 0.032 |
| LoS in patients with ischemic stroke and DM (IQR) | 4 (3-7) | 4 (3-7) | 4 (3-7) | 4 (3-7) | 4 (3-6) | 4 (3-6) | 4 (2-6) | 4 (2-6) | 4 (2-6) | 4 (2-6) | <0.001 |
| LoS in patients with TIA and DM (IQR) | 3 (2-4) | 3 (2-4) | 2 (2-4) | 2 (2-4) | 2 (2-3) | 2 (1-3) | 2 (1-3) | 2 (1-3) | 2 (1-3) | 2 (1-3) | <0.001 |

**Supplementary table 3:** Discharge disposition of patients with diabetes admitted for stroke according to stroke type. *(Numbers do not add-up due to missing data)*

| Year | 2005 | 2006 | 2007 | 2008 | 2009 | 2010 | 2011 | 2012 | 2013 | 2014 | p-trend |
| --- | --- | --- | --- | --- | --- | --- | --- | --- | --- | --- | --- |
| Hemorrhagic Stroke |  |  |  |  |  |  |  |  |  |  |  |
| Short Term | 707 (4.4%) | 889  (4.9%) | 981 (5.3%) | 1233 (6.1%) | 943 (4.5%) | 938 (4.2%) | 951 (4.5%) | 865 (4.2%) | 870 (4.1%) | 820 (3.8%) | 0.839 |
| Long term | 6678 (41.2%) | 7129 (39.6%) | 7836 (42.2%) | 8323 (40.9%) | 9047 (43.3%) | 9793 (43.7%) | 9669 (45.4%) | 9140 (44.6%) | 9180 (43.3%) | 10000 (46.1%) | 0.001 |
| Home | 1220 (7.5%) | 1275 (7.1%) | 1531 (8.2%) | 1651 (8.1%) | 1548 (7.4%) | 1795 (8.0%) | 1744 (8.2%) | 1545 (7.5%) | 1760 (8.3%) | 1805 (8.3%) | 0.004 |
| Died | 4547 (28.1%) | 5147 (28.6%) | 4569 (24.6%) | 5291 (26.0%) | 5391 (25.8%) | 5247 (23.5%) | 5001 (23.5%) | 4940 (24.1%) | 5020 (23.7%) | 5035 (23.2%) | 0.428 |
| Ischemic Stroke |  |  |  |  |  |  |  |  |  |  |  |
| Short term | 3269 (2.7%) | 4016 (3.2%) | 4388 (3.5%) | 5229 (3.8%) | 4484 (3.3%) | 4607 (3.1%) | 4692 (3.0%) | 4700 (3.0%) | 4425 (2.7%) | 4865 (2.8%) | 0.066 |
| Long term | 53678 (44.9%) | 54759 (44.2%) | 54461 (43.7%) | 59687 (43.6%) | 59171 (43.4%) | 64455 (44.0%) | 69946 (45.1%) | 69230 (44.4%) | 71595 (44.3%) | 76385 (44.5%) | <0.001 |
| Home | 14447 (12.1%) | 15872 (12.8%%) | 16019 (12.9%) | 18253 (13.3%) | 18480 (13.6%) | 20010 (13.7%) | 21458 (13.8%) | 21370 (13.7%) | 22885 (14.2%) | 24565 (14.3%) | <0.001 |
| Died | 5840 (4.9%) | 5845 (4.7%) | 5714 (4.6%) | 6532 (4.8%) | 5931 (4.4%) | 6500 (4.4%) | 6170 (4.0%) | 5810 (3.7%) | 6150 (3.8%) | 6325 (3.9%) | 0.254 |
